# Adoption of Artificial Intelligence in Rehabilitation: Perceptions, Knowledge, and Challenges Among Healthcare Providers

**DOI:** 10.3390/healthcare13040350

**Published:** 2025-02-07

**Authors:** Monira I. Aldhahi, Amal I. Alorainy, Mohamed M. Abuzaid, Awadia Gareeballah, Naifah F. Alsubaie, Anwar S. Alshamary, Zuhal Y. Hamd

**Affiliations:** 1 Department of Rehabilitation Sciences, College of Health and Rehabilitation Sciences, Princess Nourah bint Abdulrahman University, P.O. Box 84428, Riyadh 11671, Saudi Arabia; 2 Department of Radiological Sciences, College of Health and Rehabilitation Sciences, Princess Nourah bint Abdulrahman University, P.O. Box 84428, Riyadh 11671, Saudi Arabia; aialorainy@pnu.edu.sa (A.I.A.); 442001290@pnu.edu.sa (N.F.A.); zyhamd@pnu.edu.sa (Z.Y.H.); 3 Medical Diagnostic Imaging Department, College of Health Sciences, University of Sharjah, Sharjah P.O. Box 27272, United Arab Emirates; mabdelfatah@sharjah.ac.ae; 4 Research Institute for Medical and Health Sciences, University of Sharjah, Sharjah P.O. Box 27272, United Arab Emirates; 5 Department of Diagnostic Radiology, College of Applied Medical Science, Taibah University, P.O. Box 344, Al-Madinah Al-Munawwarah 41477, Saudi Arabia; agsali@taibahu.edu.sa; 6 Integrated Treatment Center, Riyadh 12281, Saudi Arabia; anwar.sla98@gmail.com

**Keywords:** AI knowledge, rehabilitation, organizational preparedness, healthcare

## Abstract

Background/Objectives: The current literature reveals a gap in understanding how rehabilitation professionals, such as physical and occupational therapists, perceive and prepare to implement artificial intelligence (AI) in their practices. Therefore, we conducted a cross-sectional observational study to assess the perceptions, knowledge, and willingness of rehabilitation healthcare providers to implement AI in practice. Methods: This study was conducted in Saudi Arabia, with data collected from 430 physical therapy professionals via an online SurveyMonkey questionnaire between January and March 2024. The survey assessed demographics, AI knowledge and skills, and perceived challenges. Data were analyzed using Statistical Package for the Social Science (SPSS 27) and DATAtab (version 2025), with frequencies, percentages, and nonparametric tests used to examine the relationships between the variables. Results: The majority of respondents (80.9%) believed that AI would be integrated into physical therapy in future, with 78.6% seeing AI as significantly impacting their work. While 61.4% thought that AI would reduce workload and enhance productivity, only 30% expressed concerns about AI endangering their profession. A lack of formal AI training has commonly been reported, with social media platforms being respondents’ primary source of AI knowledge. Despite these challenges, 85.1% expressed an eagerness to learn and use AI. Organizational preparedness was a significant barrier, with 45.6% of respondents reporting that their organizations lacked AI strategies. There were insignificant differences in the mean rank of AI perceptions or knowledge based on the gender, years of experience, and qualification degree of the respondents. Conclusions: The results demonstrated a strong interest in AI implementation in physical therapy. The majority of respondents expressed confidence in AI’s future utility and readiness to incorporate it into their practice. However, challenges, such as a lack of formal training and organizational preparedness, were identified. Overall, the findings highlight AI’s potential to revolutionize physical therapy while underscoring the necessity to address training and readiness to fully realize this potential.

## 1. Introduction

The use of artificial intelligence (AI) is increasing in various sectors, including healthcare, where it is transforming how care is delivered, managed, and optimized. It is defined by as “the science of making machines do things that would require intelligence if done by men” [1]. AI technologies offer unprecedented opportunities for enhancing patient care by providing more precise diagnostics, personalized treatment plans, and efficient rehabilitation processes. In physical therapy, AI can be used for tasks such as analyzing movement patterns, predicting recovery trajectories, and optimizing rehabilitation protocols through machine learning algorithms and wearable technologies [2,3]. The integration of AI into the healthcare system has the potential to enhance patient outcomes, streamline operations, and reduce costs [4,5]. In particular, AI applications in rehabilitation are beginning to show promise in augmenting the effectiveness and efficiency of physical and occupational therapy practices [4,6,7]. AI technologies have rapidly advanced, offering tools that can analyze vast amounts of data, support decision making, and automate routine tasks. In healthcare, AI is utilized for diagnostics, predictive analytics, personalized medicine [3], and administrative tasks [6,7]. These innovations have the potential to significantly reduce the burden on healthcare professionals, improve the accuracy of diagnoses, and provide personalized treatment plans for patients [8,9].

Within the realm of rehabilitation, AI is emerging as a powerful tool to enhance the delivery of care. AI adoption in rehabilitation is a growing trend globally, with significant advancements and applications across various domains [10]. AI-driven systems can assist in developing personalized rehabilitation programs, monitoring patient progress in real time, and predicting patient outcomes based on data patterns [7]. Moreover, AI can facilitate remote rehabilitation through virtual platforms, enabling patients to receive care outside traditional clinical settings [4,11]. In the United States, AI is being integrated into various rehabilitation programs to enhance patient outcomes and streamline healthcare processes [7]. In Europe, countries such as the United Kingdom and Germany leverage AI to support rehabilitation of neurological conditions [2,12,13,14,15]. Innovative artificial intelligence systems are being developed to support stroke patients during recovery. These advanced tools offer personalized training regimens and instantaneous guidance tailored to the specific requirements of each patient. This can lead to more consistent and accessible treatments, particularly for individuals in remote or underserved areas.

Machine learning (ML), a branch of artificial intelligence (AI), enables professionals to leverage existing data to forecast outcomes. Furthermore, ML facilitates automated decision making and generates predictions derived from patient information, serving as a valuable resource for delivering timely preventive care to individuals with health conditions. A study conducted by Ye et al. aimed to assess a tool developed using ML algorithms to evaluate fall risk in older adults. The findings indicated that the ML-driven fall risk assessment tool was effective in generating automatic early warnings, potentially reducing the incidence of falls among this demographic [16].

AI facilitates remote rehabilitation services, particularly for cardiovascular and neurodegenerative diseases, allowing for continuous monitoring and intervention from a distance [12]. Recent advancements in AI have significant potential for enhancing stroke recovery and rehabilitation by increasing the efficiency and personalization of therapeutic interventions [17]. AI applications in stroke rehabilitation primarily target areas such as motor function, gait, and mobility [18]. Advanced techniques, such as machine learning and deep learning, are employed to analyze upper limb and functional movements, often leveraging sensors such as inertial measurement units to provide detailed and precise assessments in stroke recovery and neurorehabilitation [13,15]. The development of AI-based wearable robotic exoskeletons is a significant trend that focuses on upper limb rehabilitation. These devices use artificial neural networks and adaptive algorithms to enhance the motor recovery [3].

However, despite the growing potential of AI in rehabilitation, there is a notable lack of studies exploring the perceptions and knowledge of physical and occupational therapists regarding AI in Saudi Arabia. Understanding how these professionals perceive AI, their knowledge of its application, and their willingness to incorporate it into their practice is critical. AI could significantly save time and reduce costs; however, its adoption in rehabilitation settings in Saudi Arabia may be hindered by a lack of understanding or resistance to change.

The current literature reveals a gap in understanding how rehabilitation professionals, such as physical and occupational therapists, perceive and prepare to implement AI in their practices. It remains unclear whether these healthcare providers are knowledgeable about AI technologies, how comfortable they are using them, or their willingness to accept AI as a standard component of their clinical toolkit. A study of physicians in family medicine regarding the factors influencing their adoption of AI found that performance expectancy, effort expectancy, and facilitating conditions were strong influencing factors, while regulatory support and interoperability standards were poorly rated [19]. Additionally, the study identified age-related differences in the perception of AI-enabled assistants, with younger respondents exhibiting more positive attitudes and expectations [19]. A systematic review highlighted the enablers of AI in physical rehabilitation, which included greater access to rehabilitation programs, the remote monitoring of progress, a reduction in manpower requirements, and lower costs [4]. It was concluded that the clinical effects of AI-supported physical rehabilitation interventions were inconsistent. Implementation barriers included technology literacy, reliability, and user fatigue [4].

These unknowns pose a significant challenge to the widespread adoption of AI in rehabilitation and potentially limit its impact on improving care delivery and patient outcomes in Saudi Arabia, which requires further investigation. Therefore, we conducted a cross-sectional observational study to assess the perceptions, knowledge, and willingness of rehabilitation healthcare providers to implement AI in practice. This study aimed to gather insights into how physical therapists and occupational therapists view AI, their understanding of its potential benefits and limitations, and their readiness to integrate AI into their clinical practice. The findings from this study will provide valuable information on the current state of AI adoption in rehabilitation and may inform future strategies for the education, training, and implementation of AI technologies in this field.

## 2. Materials and Methods

### 2.1. Study Design and Sampling

This cross-sectional study was conducted among physical therapy professionals working in hospitals in Saudi Arabia between January and March 2024. The sample size (n) was calculated using the formula for sample size with population (n = P (1 − P) Z^2^/d^2^), with a margin of error (d) of 0.05, a Z value of 1.96 (for a 95% confidence interval), and a population proportion of 0.5, resulting in a calculated sample size of 385 respondents. Ultimately, 430 participants agreed to participate in the study and were included in data analysis.

### 2.2. Data Collection and Instruments

The data for this study were collected using an online SurveyMonkey questionnaire, which comprised questions about the demographic information of the study participants as well as numerous closed-ended questions designed to evaluate the degree of perceptions, awareness, and willingness to adopt AI among physical therapy professionals in Saudi Arabia. The questionnaire was prepared based on previous studies [20,21] and was modified to meet the specific objectives of this study. The questionnaire consisted of three sections and ten items. The types of questions varied, including multiple-choice questions, Likert-scale items (5-point scale ranging from strongly agree to strongly disagree), and categorical questions (selecting sources of information and methods of learning AI). Section 1 was used to assess perceptions and attitudes towards AI in rehabilitation and included nine items using a Likert scale. Section 2 included items used to examine knowledge and skills related to AI, comprising a combination of multiple choice and Likert-scale items. The last section was used to assess willingness and challenges, and it included 5 questions which addressed the following concepts: organizational responsibility for AI (multiple-choice); the current role of AI in the participants’ work (multiple-choice); the presence or absence of a strategic plan for AI in their organization (multiple-choice); the biggest challenges for AI implementation (multiple-choice, participants could select multiple challenges); and the most needed AI-related applications in their work (multiple-choice with an open-ended option). To ensure content validity and relevance, a group of specialists in rehabilitation and artificial intelligence examined the questionnaires. Before distributing the survey questionnaire, a preliminary test involving 30 participants was conducted to evaluate the clarity and reliability of the questions. Reliability was evaluated using Cronbach’s alpha methods, resulting in a value of 0.780 for the perception scale and 0.736 for the knowledge and skills scale, indicating acceptable reliability and internal consistency. However, the willingness scale had a lower reliability coefficient of 0.621, suggesting that further refinement was required to improve its reliability. After refining the questions related to willingness, an online survey link was randomly distributed through social media platforms. Data collection commenced in January 2024 and continued until March 2024.

### 2.3. Ethical Approval

Prior to data collection, ethical approval was obtained from the Institutional Review Board (IRB) of Princess Nourah bint Abdulrahman University (IRB Log Number: 23-0896).

### 2.4. Data Analysis

Data analysis was performed using SPSS version 27 (IBM, Armonk, NY, USA) and the DATAtab Online Statistics Calculator (DATAtab e.U., Graz, Austria). Both descriptive and inferential statistics were employed, exploiting frequency and percentage to illustrate categorical data, such as demographic characteristics, rehabilitation professionals’ awareness and perception of AI, and sources of AI learning among study participants. For continuous variables, results were expressed as the median (MED), means and standard deviations (mean ± SD), and the mode for each question within each domain, and then the weighted average for each domain was calculated. Any mean value in each of the questions used to assess the participants’ knowledge, awareness, and perception, if it was lower than the weighted average, was considered to represent high perception, awareness, and knowledge, and any mean values that were higher than the weighted average were considered to represent low perception, awareness, and knowledge. Categorical variables are presented as frequencies and percentages (%). Kolmogorov–Smirnov and Shapiro–Wilk tests were conducted to determine the normality of the data prior to analysis. The results revealed that the mean scores for all domains were not normally distributed, with *p*-values indicating significant differences (Kolmogorov–Smirnov, *p* < 0.001; Shapiro–Wilk, *p* < 0.001) across all domains. Kruskal–Wallis and Mann–Whitney tests were used to compare the mean scores of perceptions and knowledge based on the participants’ demographic factors (age, gender, years of experience, and qualification levels). Differences were considered statistically significant at an alpha level of 0.05.

## 3. Results

### 3.1. Demographic Data

Table 1 presents the analyzed responses of the 430 respondents about their demographic characteristics. Most respondents were female (62.8%, n = 270) and in the 20–30-year-old age category (75.8%, n = 326). Most participants held a B.Sc. degree (80.7%, n = 347), with a significant proportion obtaining qualifications from Saudi Arabia (93.5%, n = 402). Those who worked in Saudi Arabia totaled 97.9% (n = 421), most respondents had less than 10 years of experience (91.9%, n = 395), and most respondents worked in a governmental hospital (72.8%, n = 313).

### 3.2. Perceptions and Attitudes Toward AI in Physical Therapy

Regarding the perception and attitude of the study participants toward AI, the results demonstrated that the majority (78.6%) believed that AI will play an important role in their professions, with an average score of 1.89 (SD 0.88). Most respondents (80.9%) agreed that AI will be applied in physical therapy in future, with an average score of 1.88 (SD 0.84). Only 30% strongly agreed or agreed that AI will threaten PT professionals, while 43% disagreed or strongly disagreed, showing more neutral to negative responses (mean 3.09, SD 1.21). Similarly, concerns were stated about AI disrupting the careers of some PT professionals, with 31.6% agreeing and 38.8% disagreeing, with a mean score of 3.04 (SD 1.18). While 45.6% agreed or strongly agreed that AI has no limitations, 34% disagreed or strongly disagreed, indicating uncertainty that AI may cause a limitation in their work (mean 2.93, SD 1.16). Over 60% of respondents (61.4% and 76.3%, respectively) believed AI can reduce workload and increase productivity; the mean scores were 2.40 (SD 0.99) for workload reduction and 2.02 (SD 0.85) for productivity improvement, respectively. Most of the study participants (75.8%) felt that AI would improve their quality of life (average: 2.04, SD: 0.87). The participants’ responses demonstrated high openness among professionals to adopt AI in their work, with 85.1% voicing agreement in their readiness to learn and adopt AI in practice and only 1.8% disagreeing (mean 1.74, SD 0.82) (Table 2).

Table 3 shows various sentiments among the study participants concerning the integration of AI into physical therapy; 47.9% demonstrated excitement about the role of AI in their field, 21.9% described that they were aware of the challenges that AI may introduce, and 20.2% of the participants stated that they neither reinforced nor opposed AI’s integration in their practice. Fewer participants (2.1%) felt overwhelmed and uncertain about keeping up with AI advancements, while 2.3% stated they were concerned about the impact of AI on their profession, and 5.6% mentioned that they did not know enough about AI.

### 3.3. Knowledge and Skills in AI for Physical Therapy Professionals

The result of the study demonstrated that most respondents believed that AI should be part of the PT curriculum (28.4% strongly agree, 42.6% agree), while a minimal number of respondents disagreed that AI be a part of the curriculum (5.8%), with an average score of 2.07 (SD 0.87). Furthermore, 25.1% strongly agreed and 43.3% agreed that AI should be taught in undergraduate programs; the mean score for this item was 2.15 (SD 0.89). Similarly, 27.7% strongly agreed and 42.1% agreed that AI should be taught in postgraduate programs, with an average score of 2.08 (SD 0.86). The results demonstrated a gap in the respondents’ understanding and practical knowledge of AI: while 12.3% strongly agreed and 33.3% agreed that they have a basic understanding of AI relevant to their field, 19.3% disagreed, indicating that many lack confidence in their knowledge. Furthermore, only 10.9% strongly agreed and 24.4% agreed that they possess a working knowledge of AI, with a notable 29.5% strongly disagreeing. The most outstanding finding among the respondents is their lack of formal AI training, as only 7.9% strongly agreed and 15.6% agreed that they have been trained or educated in AI. A substantial 37.4% strongly disagreed, with an average score of 3.41 (SD 1.1), which was greater than the weighted average, thereby indicating a significant gap in AI education (Table 4)

**Figure 1:**
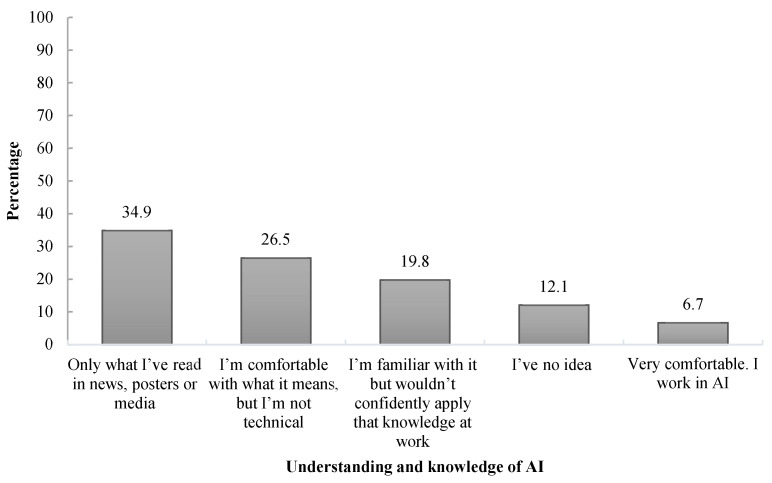
Knowledge of AI among the study participants.

Further, participants were asked how well they understood what was meant by AI. A majority, 34.9% (n = 150), indicated that their understanding came from what they had previously read in news, posters, or media, while 26.5% mentioned that they were comfortable what AI meant but not technically; furthermore, 19.3% mentioned that they were familiar with AI, but not confident in applying it in their works (Figure 1).

**Figure 2:**
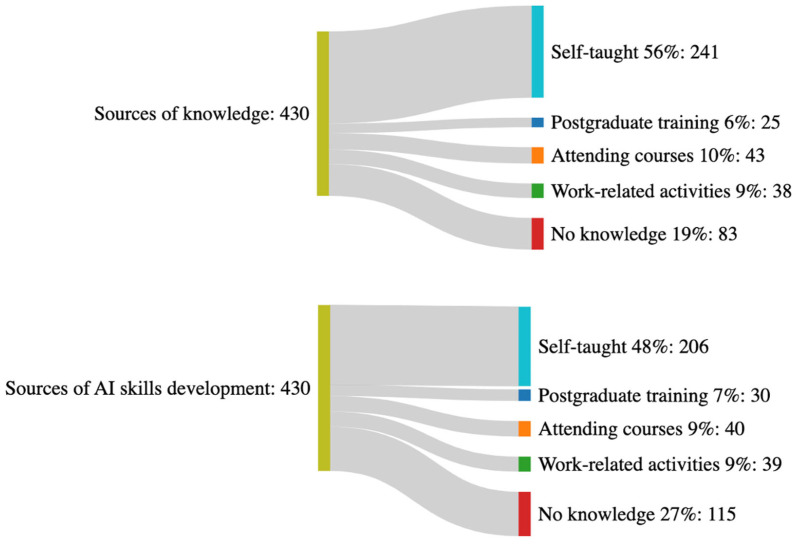
Source of knowledge and development of AI skills among the study participants.

The Sankey diagram in Figure 2 demonstrates the source of knowledge and development of AI skills. The majority, 56% (n = 241), reported that their knowledge of AI was self-taught, whereas 19% (n = 83) indicated that they do not know AI. Regarding how their skills in AI developed, 47.9% (n = 206) indicated their skills were self-taught, while 26.7% (n = 115) stated they do not know AI (Figure 2).

**Figure 3:**
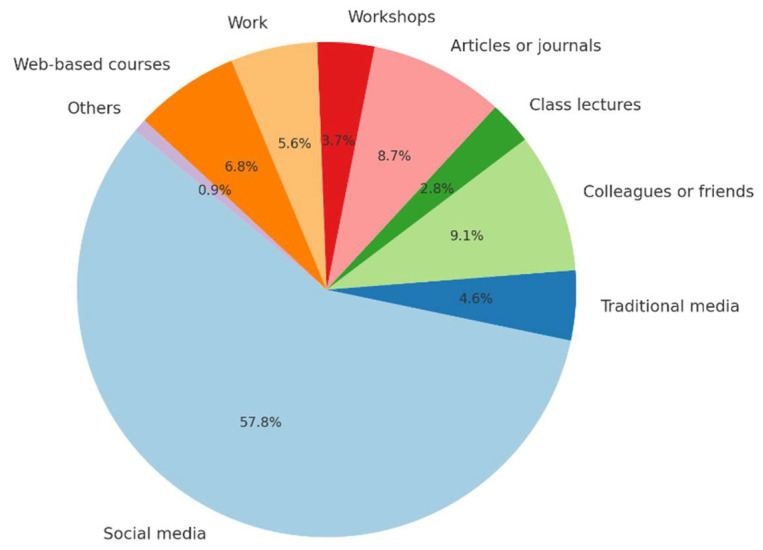
The sources of AI information among respondents.

Figure 3 illustrates the sources of AI information among the respondents; the majority (59.6%, n = 265) reported that social media platforms were their primary source of AI information, followed by colleagues or friends (9.4%, n = 42), articles or journals (9%, n = 40), and web-based courses (7%, n = 31).

### 3.4. Willingness to Adopt AI, Organizational Readiness, and Key Implementation Challenges

Most respondents indicated that their organizations do not have an employee responsible for AI, with 45.6% (n = 196) responding “No”. When asked about the role of AI in their work, the largest group, 30.5% (n = 131), stated that they had no idea about AI’s role in their work. Regarding their work organization’s strategy for AI, the majority, 42.1% (n = 181), indicated that they had no idea if a strategy was in place. Finally, the biggest challenge for AI implementation in their work was identified as a ‘lack of knowledge’, with 22.8% (n = 98) of respondents highlighting this issue (Table 5).

**Figure 4:**
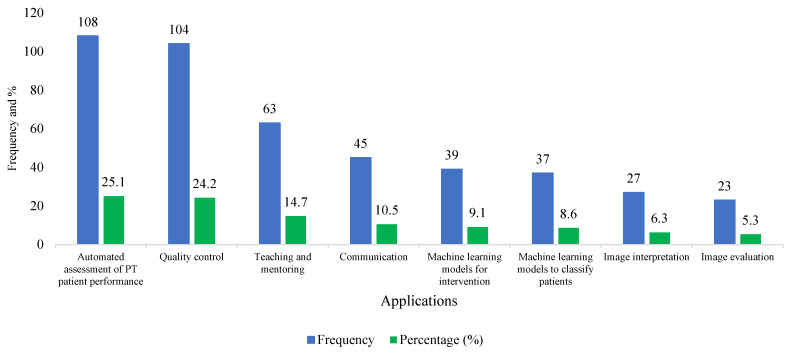
The bar chart shows the application of AI in PT practice.

The graph in Figure 4 illustrates the application of AI in the respondents’ work, with most of them mentioning the automated assessment of PT patient performance (25.1%) and quality control (24.2%), followed by teaching and mentoring (14.7%).

**Figure 5:**
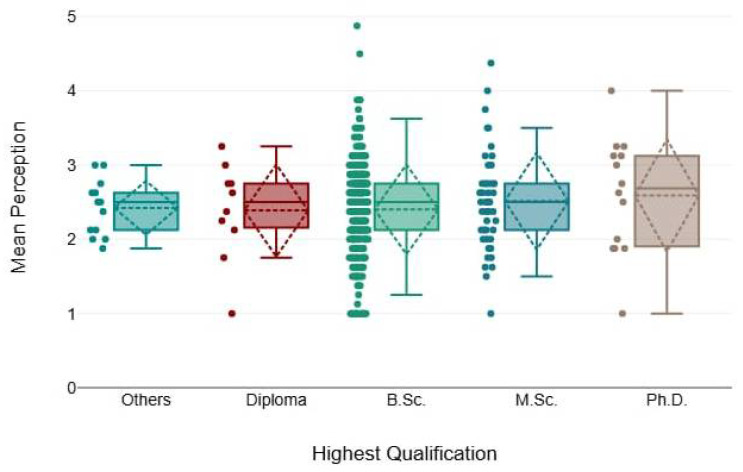
The mean perception scores among different qualification levels.

**Figure 6:**
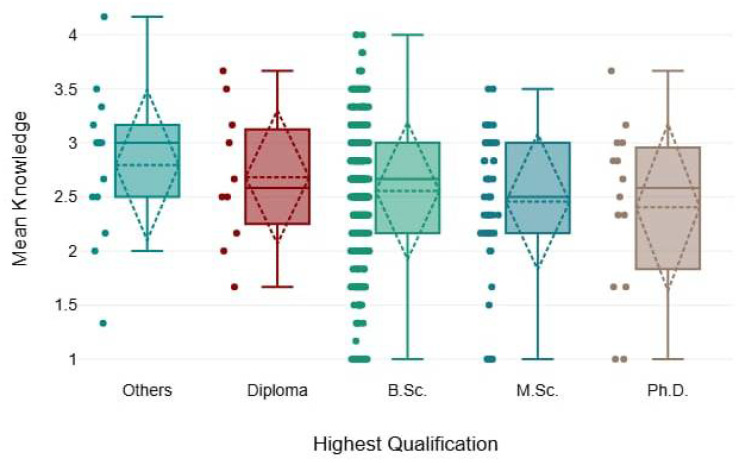
The mean knowledge scores among different qualification levels.

In Table 6, there were insignificant differences in the mean rank of knowledge and perception across different qualification levels, as the chi-squared test resulted in smaller values (1.59 and 2.9) and *p*-values were >0.05, as demonstrated below (Figure 5 and Figure 6).

The Mann–Whitney U test results suggest that there is a very small effect size (r) of the difference in mean perception and knowledge scores between males and females, with the male group being more likely to have higher mean perception scores and lower mean knowledge scores. For years of experience, the effect size was very small, indicating insignificant differences between knowledge and perception scores for those with less than and those with more than 10 years of experience; respondents with less than 10 years’ experience demonstrated higher knowledge scores and lower perception scores, but these differences were not statistically significant at the 5% significance level (Table 7).

## 4. Discussion

In rehabilitation science, the integration of AI has transformed patient care, therapy customization, injury prevention, and rehabilitation methods. AI technologies, including machine learning and deep learning, have introduced precision and efficiency into rehabilitation procedures, leading to improved patient outcomes and recovery processes. For example, deep learning algorithms can analyze motion data from sensors or imaging modalities, such as motion capture systems, accelerometers, or cameras, and can identify patterns related to movement disorders, gait abnormalities, or motor function impairments [22]. Artificial intelligence in physical therapy has great potential to improve clinical outcomes, streamline administrative procedures, and improve patient care [4]. Nonetheless, the level of eagerness for AI adoption among physical therapists and healthcare organizations varies significantly. This study aimed to evaluate the current level of awareness, perceptions, and acceptance of AI among physical therapists in Saudi Arabia, providing insight into the readiness for AI adoption in this field. The results of the study revealed the strong feelings of optimism and openness to the integration of AI within the physical therapy profession.

The results demonstrated a generally optimistic attitude among physical therapy professionals toward the implementation of AI in their field. Most participants believed that AI will play an imperative role in their profession and will be useful in physical therapy in future, reflecting strong confidence in its potential benefits. Similarly to this study, Abuzaid et al. found that most respondents agreed that AI would play a crucial role in the practice of physiotherapy professionals [21]. Worries about AI threatening or disrupting careers were present but not dominant, with more respondents giving neutral than negative responses.

While some respondents expressed concerns about AI’s limitations, studies have demonstrated its positive impact on healthcare. Our research participants noted AI’s ability to reduce workloads and enhance both patient care and operational efficiency. These findings align with Alsobhi et al.’s conclusions regarding AI’s benefits to productivity and resource optimization [20]. In another qualitative study, most interviewees agreed that artificial intelligence would not replace therapy professionals, highlighting the critical role of interpersonal interactions as essential for the safe and effective provision of vital services, including healthcare. They also mentioned that many rehabilitation services rely on manual skills and techniques applied by therapists. Nevertheless, a consensus emerged among participants that AI has the potential to alleviate the physical demands and daily workload of physical therapists [23]. By diminishing their workload, an effective work environment can be fostered, leading to enhanced job satisfaction for therapists. Furthermore, the study demonstrated participants’ substantial readiness to learn and adopt AI, indicating a high level of openness among professionals towards integrating AI into their PT practice. Similarly to our findings, Abuzaid et al. reported that approximately 83.4% of physical therapists stated that they were ready to learn and apply AI in practice [21].

This study found that, while many participants were enthusiastic about the integration of AI into physical therapy, others acknowledged potential challenges. Some felt overwhelmed by AI advancements, while others were concerned about their impact on their profession. A minority also expressed a lack of knowledge of AI. In this study, most respondents supported the inclusion of AI in physical therapy education; it is known that AI can enhance access to information, increase productivity, and reduce errors [24]. However, some respondents expressed concerns about potential challenges such as the risk of false information and plagiarism. Nevertheless, existing tools and strategies can effectively address and mitigate these issues.

The most outstanding finding was the lack of formal AI training, as a considerable number of the respondents reported no education or training in AI, demonstrating a clear need for enhanced AI education and training within the field. A substantial knowledge gap exists in AI comprehension and application among healthcare practitioners. Although some participants demonstrated fundamental AI literacy, many reported low confidence in their understanding, with only a minority claiming practical expertise. Clinical exposure is a critical factor motivating healthcare professionals to implement AI solutions in their practice. These findings underscore the importance of targeted AI education through specialized training programs and workshops for physical therapists to accelerate implementation and acceptance. Recent studies indicate growing attention to the incorporation of AI into the realm of physical therapy; however, a considerable knowledge gap persists among practitioners. Physical therapists generally acknowledge the potential advantages of AI in their work; however, they face challenges such as a lack of educational resources and training opportunities [20,21]. The biggest challenge identified for AI execution in practice was the lack of knowledge and skills, which was the most frequently mentioned issue. Prior research has identified several significant barriers to the implementation of AI in healthcare settings. These obstacles encompass insufficient domain expertise, ethical considerations, AI’s limited capability to address comprehensive patient conditions, financial constraints, and inadequate technological infrastructure [21,24].

Participants were queried about their understanding of AI, and the majority indicated that their knowledge primarily comes from media sources, such as news, posters, or other forms of media. A smaller group felt comfortable with the concept of AI but lacked technical experience, whereas others were acquainted with AI but not confident in applying it to their work. In terms of how participants attained AI knowledge and developed related skills, the majority reported that their knowledge was self-taught, and a significant number stated that they did not possess AI skills. This highlights a reliance on informal learning and a notable gap in formal AI education and skill development among the participants. Similarly to our findings, Hamd et al. [25] identified self-directed learning as the predominant method of acquiring AI knowledge and related skills among radiography professionals.

Most respondents indicated that their organizations did not have a designated person responsible for AI implementation. When asked about the role of AI in their work, a significant portion expressed uncertainty, with many stating that they had no idea about the role of AI in their workplace. Similarly, when asked about their organization’s AI strategy, the majority were unsure whether such a strategy existed. Similarly, Abuzaid et al. clarified that there were no personnel responsible for AI and integration in practice, and 45.8% of the participants stated that they expected that their organization would have a strategy for AI in the future [21]. In another study, only 5% of the respondents indicated having hands-on experience with AI applications in professional settings [24]. This finding aligns with earlier studies, which revealed that fewer than 9.5% of surgeons apply AI techniques in emergency and trauma surgery, whereas 60% reported a lack of AI and robotic technologies in their clinical practice [26].

Artificial intelligence is transforming data science and information technology through the enhancement of automated tasking technologies. Its applications are increasing across diverse healthcare sectors and industries, yielding numerous advantages, such as expedited healthcare delivery and diminished workload. For example, the literature indicates that artificial intelligence assists healthcare professionals with patient record management and the administration of medical diagnoses and treatments for various diseases while also augmenting human decision-making and operational efficiency [27]. Regarding AI applications in PT practice, respondents mostly mentioned the automated assessment of patient performance and quality control as key applications, followed by teaching and mentoring, as well as machine learning for interventions and the classification of patients. Several studies have reported on the applications of AI in physical therapy. Tack et al. mentioned applications such as automated tasks involving data analysis, classification, and prediction [27]. Rathod et al. stated that AI applications in physiotherapy include assisting in movement evaluation, rehabilitation program design, and patient monitoring by investigating innumerable data inputs to afford tailored treatment strategies [27]. Another study concluded that AI technologies are being used to transform various aspects of physiotherapy, including the assessment, diagnosis, treatment, and management of neurological and musculoskeletal conditions [28].

There were no statistically significant differences in knowledge about and perceptions of AI across different qualification levels. Gender differences showed that males tended to have higher knowledge scores but lower perception scores than females. Experience levels also showed minimal impact, with those with less than 10 years of experience demonstrating higher perception scores, although the differences were not statistically significant. Inconsistent with this study, several studies in the literature found that demographic characteristics such as gender, professional experience, and educational background significantly influenced AI knowledge and perception [20,24].

This study provides important insights into the incorporation of artificial intelligence within the realm of physical therapy, highlighting the importance of physiotherapy professionals adopting AI in their practice and the necessity for education and training. Furthermore, the results verified that the presence of AI in educational programs and training is crucial for developing practical skills and bridging the gaps. To address the identified challenges, such as the lack of training and organizational unpreparedness, several targeted plans and policy recommendations can be proposed. Educational institutions should introduce structured AI curricula to both undergraduate and postgraduate programs. This would guarantee that students graduate with the skills required to successfully incorporate AI into their professional work. Additionally, collaboration among policymakers, professional organizations, and AI developers can tackle organizational gaps by establishing guidelines, sharing best practices, and creating funding opportunities [29]. Implementing these approaches can address obstacles and promote the long-term incorporation of artificial intelligence into rehabilitation services.

This study has several limitations that warrant acknowledgment. While this study provides valuable insights into the adoption of artificial intelligence in rehabilitation among healthcare providers in Saudi Arabia, the findings may not be fully generalizable to other countries or regions owing to cultural, educational, and systemic differences in healthcare delivery. Although the sample size was substantial, it may not fully capture the perspectives of all physical therapy professionals in Saudi Arabia, potentially limiting the generalizability of the findings. This study had additional limitations, including potential self-selection bias resulting from the use of the non-probability sampling method. This approach may have attracted participants who were already interested in artificial intelligence. Consequently, the sample may overrepresent professionals who are more technologically adept or actively engaged with digital tools in their work. Moreover, the cross-sectional design of the study precludes an assessment of the long-term impact of AI education and training, underscoring the need for longitudinal research to better understand the sustained outcomes and trends over time. Additionally, the regional focus on Saudi Arabia may limit the applicability of the findings to other geographic or cultural contexts. Furthermore, the broad scope of the survey may have overlooked specific nuances related to different AI tools and the unique challenges and benefits associated with each. These factors should be considered when interpreting the results and planning future investigations.

## 5. Conclusions

The study concluded that physical therapy professionals in Saudi Arabia are eager to integrate artificial intelligence into their practices, despite the potential challenges encountered, such as insufficient training, limited AI experience, and a lack of knowledge. They deemed that AI has the potential to enhance productivity, improve the quality of patients’ lives, and reduce their workload. Insignificant differences in the degree of knowledge, awareness, and perception of AI based on education level, experience, and gender were noted. To improve AI education and training, it is crucial to integrate AI-related content into professional development and academic curricula. Moreover, workshops, seminars, and awareness creativity should be encouraged to educate PTs on the practical applications of AI. Institutions should also develop supportive resource and technological frameworks for AI implementation.

## Figures and Tables

**Table 1:** The frequency distributions of demographic characteristics of the study participants.

Demographic Characteristics		n	%
Gender	Female	270	62.8
	Male	160	37.2
Age category	20–30	326	75.8
	31–40	92	21.4
	41–50	10	2.3
	Older than 50	2	0.5
Highest qualification	Other	13	3
	Diploma	10	2.3
	B.Sc.	347	80.7
	M.Sc.	46	10.7
	Ph.D.	14	3.3
Country from which qualification was awarded	KSA	402	93.5
	Other	28	6.5
Country in which participant is currently working	KSA	421	97.9
	Other	9	2.1
Work experience	≤10 years	395	91.9
	>10 years	35	8.1
Work settings	Governmental hospital	313	72.8
	Clinics and health centers	117	27.2

**Table 2:** Perceptions and attitudes of the respondents concerning AI use in physical therapy.

Items	Response (n, %)					Mean	SD	Median (Mode)	Decision
	SA	AG	Neu	DA	SDA				
AI will play an important role in the practice of PT professions.	165 (38.4%)	173 (40.2%)	70 (16.3%)	19 (4.4%)	3 (0.7%)	1.89	0.88	2 (2)	High
AI will take place in many applications.	154 (35.8%)	194 (45.1%)	60 (14%)	20 (4.7%)	2 (0.5%)	1.88	0.84	2 (2)	High
AI will threaten/disrupt the PT professions’ practice.	53 (12.3%)	76 (17.7%)	116 (27%)	135 (31.4%)	50 (11.6%)	3.09	1.21	3 (4)	Low
AI will threaten/disrupt the careers of some professionals in the PT field.	49 (11.4%)	87 (20.2%)	127 (29.5%)	120 (27.9%)	47 (10.9%)	3.04	1.18	3 (3)	Low
AI has no limitations in my work.	58 (13.5%)	88 (20.5%)	138 (32.1%)	110 (25.6%)	36 (8.4%)	2.93	1.16	3 (3)	Low
AI will reduce PT workload.	73 (17%)	190 (44.4%)	106 (24.7%)	49 (11.4%)	12 (2.8%)	2.40	0.99	2 (2)	Low
AI will improve patients’ quality of life.	118 (27.4%)	208 (48.4%)	78 (18.1%)	21 (4.9%)	5 (1.2%)	2.04	0.87	2 (2)	High
AI will increase PT productivity.	122 (28.4%)	206 (47.9%)	81 (18.8%)	16 (3.7%)	5 (1.2%)	2.02	0.85	2 (2)	High
I am ready to learn and apply AI in my practice.	194 (45.1%)	172 (40%)	53 (12.3%)	7 (1.6%)	4 (0.9%)	1.74	0.82	2 (1)	High

Note: Data presented as frequency (%). Abbreviations: SA (strongly agree), AG (agree), Neu (neutral), DA (disagree), SDA (strongly disagree), PT (physiotherapist). Weighted average = 2.34. Decision based on the weighted average; if the mean in each question is lower than the weighted average, it is considered to represent high perception, and if it is higher than the weighted average, it is considered to represent low perception.

**Table 3:** Participants’ perspectives on the integration of AI in physical therapy.

Items	Frequency (n)	Percentage (%)
Excited about the integration of AI in the field	206	47.9
Aware of the challenges of AI implementation and usage	94	21.9
Neither agree nor disagree with integration of AI in the field	87	20.2
I do not know enough about AI	24	5.6
Worried about the impact on the practice	10	2.3
Overwhelmed, I do not feel I can keep up	9	2.1
Total	430	100

**Table 4:** Respondents’ knowledge and skills in relation to AI integration in physical therapy.

Items	Response, n (%)					Mean	SD	Median (Mode)	Decision
	SA	AG	Neu	DA	SDA				
All rehabilitation professions’ curricula should include at least some basic knowledge of AI	122 (28.4)	183 (42.6)	99 (23)	25 (5.8)	1 (0.2)	2.07	0.87	2 (2)	High
AI should be taught in undergraduate programs	108 (25.1)	186 (43.3)	104 (24.2)	29 (6.7)	3 (0.7)	2.15	0.89	2 (2)	High
AI should be taught in postgraduate programs	119 (27.7)	181 (42.1)	108 (25.1)	21 (4.9)	1 (0.2)	2.08	0.86	2 (2)	High
I have a basic understanding of AI (relevant to my field).	53 (12.3)	143 (33.3)	143 (33.3)	83 (19.3)	8 (1.9)	2.64	0.99	3 (2)	Low
I have a working knowledge of AI (relevant to my field).	47 (10.9)	105 (24.4)	133 (30.9)	127 (29.5)	18 (4.2)	2.90	1.07	3 (3)	Low
I have been trained and educated about AI relevant to my field	34 (7.9)	67 (15.6)	86 (20)	161 (37.4)	82 (19.1)	3.40	1.2	3 (4)	Low

Abbreviations: SA (strongly agree), AG (agree), Neu (neutral), DA (disagree), SDA (strongly disagree). Data are presented as frequency (percentage); weighted average = 2.55. Decision is based on the weighted average; if the mean in each question is lower than the weighted average, it is considered to represent high knowledge and, if the mean is higher than the weighted average, it is considered to represent low knowledge and skills.

**Table 5:** Willingness to adopt AI, organizational readiness, and key implementation challenges.

Items	Responses	n	%
In the organization, there is an individual responsible for AI	Yes.	69	16
	No.	169	45.6
	I am not sure.	165	38.4
The role of AI in the work is defined	It is a major component.	36	8.4
	It plays a minor role.	65	15.1
	We are just getting started.	51	11.9
	It is part of our future plans.	88	20.5
	We are not currently considering or planning for AI.	59	13.7
	I’m unsure about AI’s relevance to my work.	131	30.5
The main challenges for AI execution in your work	A lack of knowledge.	98	22.8
	Developing skills.	97	22.6
	It is hard to find good education and training courses in AI.	72	16.7
	It is hard to educate and train the current staff in AI technology.	47	10.9
	It is hard to implement AI in our work and practice.	68	15.8
	Graduates are not learning AI knowledge and skills at university.	48	11.2
My work organization has a strategy for AI.	Yes.	59	13.7
	No.	157	36.5
	We are developing one.	33	7.7
	I have no idea.	181	42.1

**Table 6:** Impact of qualification level in knowledge and perception scores.

Domains	Groups	n	Median	Mean Rank	Chi^2^	*p*-Value
Perception score	B.Sc.	347	2.5	213.05	1.59	0.811
	M.Sc.	46	2.5	223.57		
	Ph.D.	14	2.69	252.11		
	Other	13	2.5	209.96		
	Diploma	10	2.5	219.25		
	Total	430	2.5			
Knowledge score	B.Sc.	347	2.67	216.12	2.9	0.575
	M.Sc.	46	2.5	199.62		
	Ph.D.	14	2.58	197.86		
	Other	13	3	258.77		
	Diploma	10	2.58	235.5		
	Total	430	2.67			

**Table 7:** Impact of gender and years of experience on knowledge and perception scores.

Variables		n	Mean Rank	Mean (Median)	U	z	*p*-Value	r
Knowledge score	Male	160	209.7	2.52 (2.5)	20,671.5	−0.75	0.454	0.04
	Female	270	218.94	2.57 (2.67)				
	<10 years	395	215.91	2.55 (2.67)	6752	−0.23	0.82	0.01
	>10 years	35	210.91	2.55 (2.5)				
Perception score	Male	160	226.79	2.47 (2.5)	19,794	−1.45	0.147	0.07
	Female	270	208.81	2.39 (2.5)				
	<10 years	395	212.66	2.41 (2.5)	5789	−1.6	0.111	0.08
	>10 years	35	247.6	2.56 (2.63)				

## Data Availability

The datasets used and/or analyzed during the current study are available from the corresponding author on reasonable request.
